# Tree-ring data set for dendroclimatic reconstructions and dendrochronological dating in European Russia

**DOI:** 10.1038/s41597-022-01456-6

**Published:** 2022-06-27

**Authors:** Olga Solomina, Vladimir Matskovsky, Ekaterina Dolgova, Veronika Kuznetsova, Nadezhda Semenyak, Tatiana Bebchuk, Vladimir Mikhalenko, Alexey Karpukhin, Bulat Khasanov

**Affiliations:** 1 Institute of Geography RAS, Moscow, Russia; 2 HSE University, Moscow, Russia; 3 Institute of Archaeology RAS, Moscow, Russia; 4 A.N. Severtsov’s Institute of Ecology and Evolution RAS, Moscow, Russia

**Keywords:** Palaeoclimate, Forest ecology

## Abstract

The data set presented represents 15 years of collection. It contains tree-ring width measurements from 64 sites of living trees and ten historical chronologies based on archaeological and construction wood up to year 572 CE, altogether 2909 tree-ring series and more than 450000 measured and cross-dated tree rings. It covers the vast territory of European Russia, including its forested northern and central parts, and the Northern Caucasus mountains. The potential use of these data include climatic reconstructions of regional and hemispheric scale, dendrochronological dating of historical and cultural wood, ecological and remote sensing studies.

## Abstract (summary)

**Table:** 

Measurement(s)	growth ring
Technology Type(s)	Measurement Device
Factor Type(s)	location
Sample Characteristic - Organism	Pinus sylvestris • Picea abies • Quercus robur • Larix sibirica • Fagus orientalis
Sample Characteristic - Environment	woodland area
Sample Characteristic - Location	European Russia

## Background & Summary

Tree rings are an important source of ecological, biological, paleoclimatic, astronomic, geomorphic, archaeological, and cultural information^1,2^. In dendrochronology various parameters of tree rings are used, such as total ring width, early and late wood ring width, density, cell wall and cell lumen area, stable isotopes of different elements, chemical composition as well as specific features of the wood (tension and compressive wood, resin ducts, light and frost rings, fire scars, etc.)^2–5^. However, the tree-ring width is the basic parameter that serves to establish the chronology before all other parameters can be measured. In some regions, for example in the North America and Western Europe, tree-ring chronologies are abundant and are represented in the databases with open access. The most comprehensive global tree-ring database is the International Tree-Ring Data Bank^6^ (ITRDB, https://www.ncei.noaa.gov/products/paleoclimatology/tree-ring) that includes several thousand of entries in standard format. The tree-ring data from the ITRDB are intensively used in various studies, including large-scale regional and global palaeoreconstructions^7^.

Tree-ring data from European Russia is represented rather poorly in the ITRDB so far. Most of those records are from living trees concentrated along the northern tree line and predominantly come from the massive sampling campaigns that occurred in the end of 1990s^8,9^, and a recently submitted collection of 13 chronologies from the central and northern parts of the region^10^. In the 2000s several regional wood collections appeared in the Volga region^11^, in Voronezh region^12^, in the Komi Republic^13^ etc., although they are not in the ITRDB. The number of chronologies based on sub-fossil and archaeological wood and submitted to the ITRDB is even smaller^14,15^. These last two types of chronologies are not connected to any living-tree chronology from nearby locations. A more detailed description of the current state of dendrochronological data and research in the region may be found in the recent review^16^.

In this paper we introduce a collection of 64 ring-width chronologies from living trees for European Russia, including Northern Caucasus: pine (*Pinus sylvestris* L.), spruce (*Picea abies* (L.) Karst.*, P.obovata* Lebed.), Larch (*Larix sibirica* Lebed.), and oak (*Quercus robur* R.). Beech (*Fagus orientalis* Lipsky) and fir (*Abies nordmanniana* (Stev.) Spach) – are represented by single living tree chronologies. Unlike the East European plain the tree-ring sites at the Northern Caucasus are located predomonantly at the high elevation^17^. Most of the samples were collected at the upper tree limit. Some as high as the Little Ice Age moraines to date the glacier advances^18^, and some at lower elevations, in the middle of the forest. The natural, drought-sensitive, lower tree limit in the Caucasus has risen by centuries of logging leaving very few opportunities to find long-living trees suitable for dendroclimatic reconstructions and dendrochronological dating.

Moreover, we introduce ten absolutely dated regional conifer and oak chronologies based on archaeological, architectural, and subfossil wood. The wood mostly comes from the old buildings. Some of the buildings have been or are being renovated with the replacement of wooden elements; others are currently crumbling. Many architectural monuments from which we collected wood for the chronologies presented here either do not exist anymore or were totally renovated with all the ancient wood replaced and thus forever lost for scientific use.

**Fig. 1:**
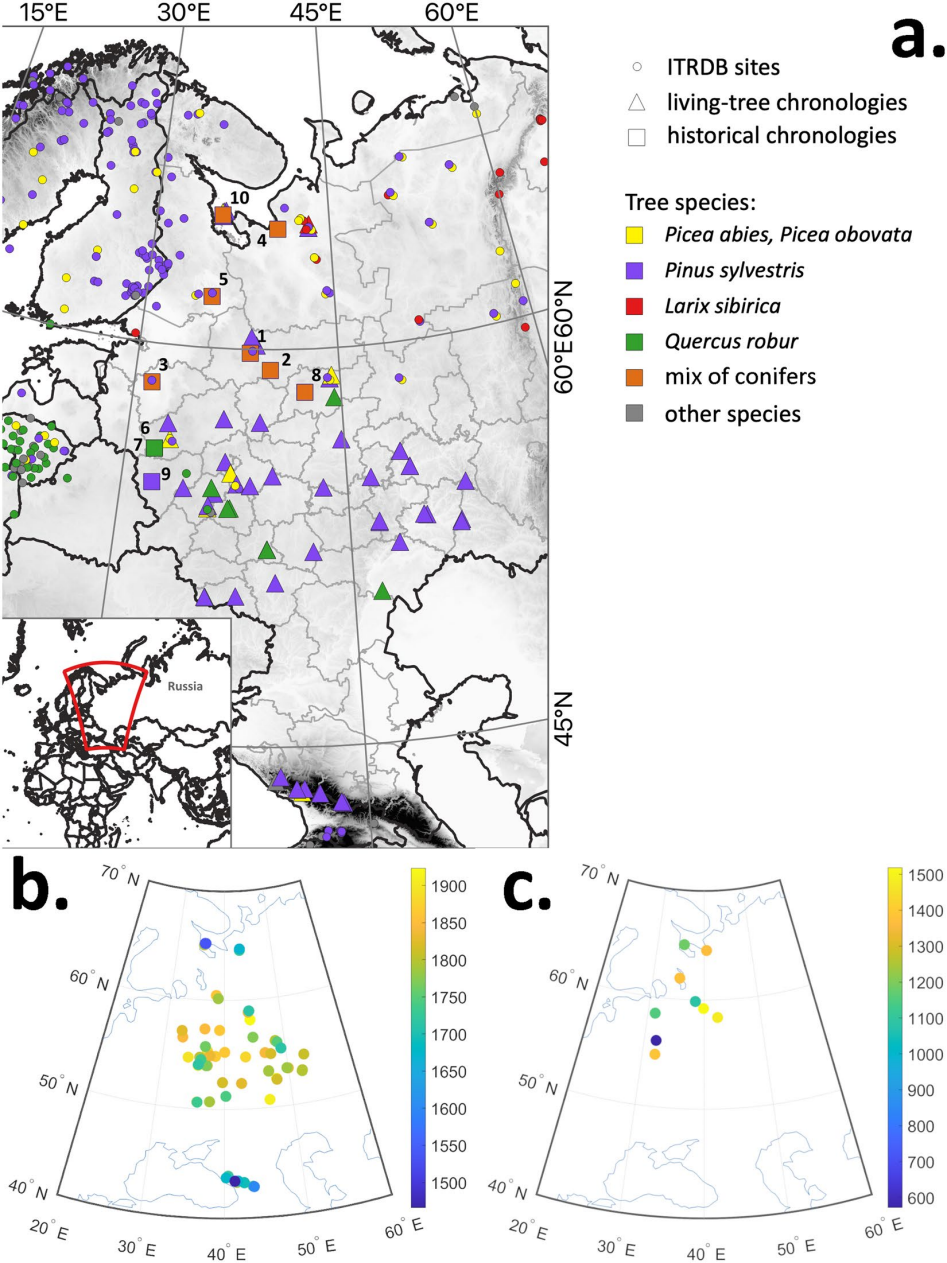
(**a**) Map of the study region with the chronologies represented in ITRDB and in the described data set. Historical chronologies are: 1 Kirillov, 2 Vologda, 3 Novgorod, 4 Arkhangelsk, 5 Karelia, 6 Zapadnaya Dvina 1, 7 Zapadnaya Dvina 2, 8 Kostroma, 9 Smolensk, 10 Solovki. Grayscale shading shows elevation. (**b**) Map with the locations of the ‘living-tree’ chronologies. The first year of the chronologies is shown by color. (**c**) Map with the locations of the ‘historical’ chronologies. The first year of the chronologies is shown by color. Note different color scales in (**b**) and (**c**).

**Fig. 2:**
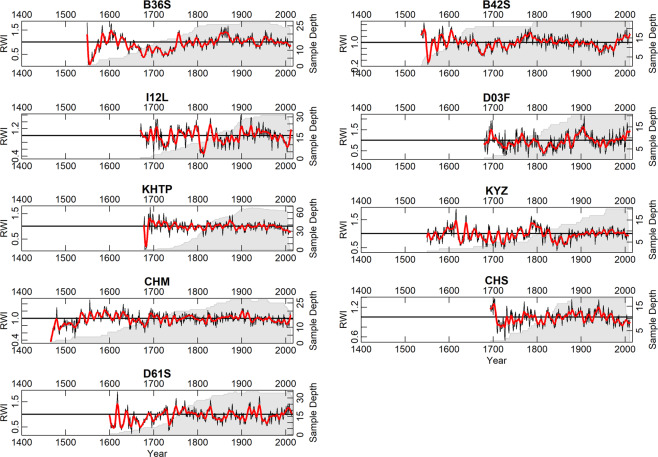
The longest ‘living-tree’ chronologies: those that start before CE 1700. Black lines show tree-ring indices produced by ‘ModNegExp’ option in ‘detrend’ function of R package dplR^49^. Red lines show 11-yr smoothing splines fitted to the tree-ring indices. Grey shading show sample depth – the number of measurements in each year. As in online-only Tables 1 and 2, the chronologies are represented from North to South.

The wood in the collection described herein has been collected over the previous 15 years. The collection consists of 74 sites with 1680 trees and 2909 individual tree-ring series, and 456984 measured tree rings (Fig. 1). Most of the wood in the collection is available in the Institute of Geography RAS and accessible for further investigations upon request. Some scientific results based on these data are presented elsewhere^17–22^. The chronologies are plotted in Fig. 2 (the longest ‘living-tree’ chronologies, those that start before CE 1700), Fig. 3 (all the other living-tree’ chronologies), and Fig. 4 (historical chronologies).

**Fig. 3:**
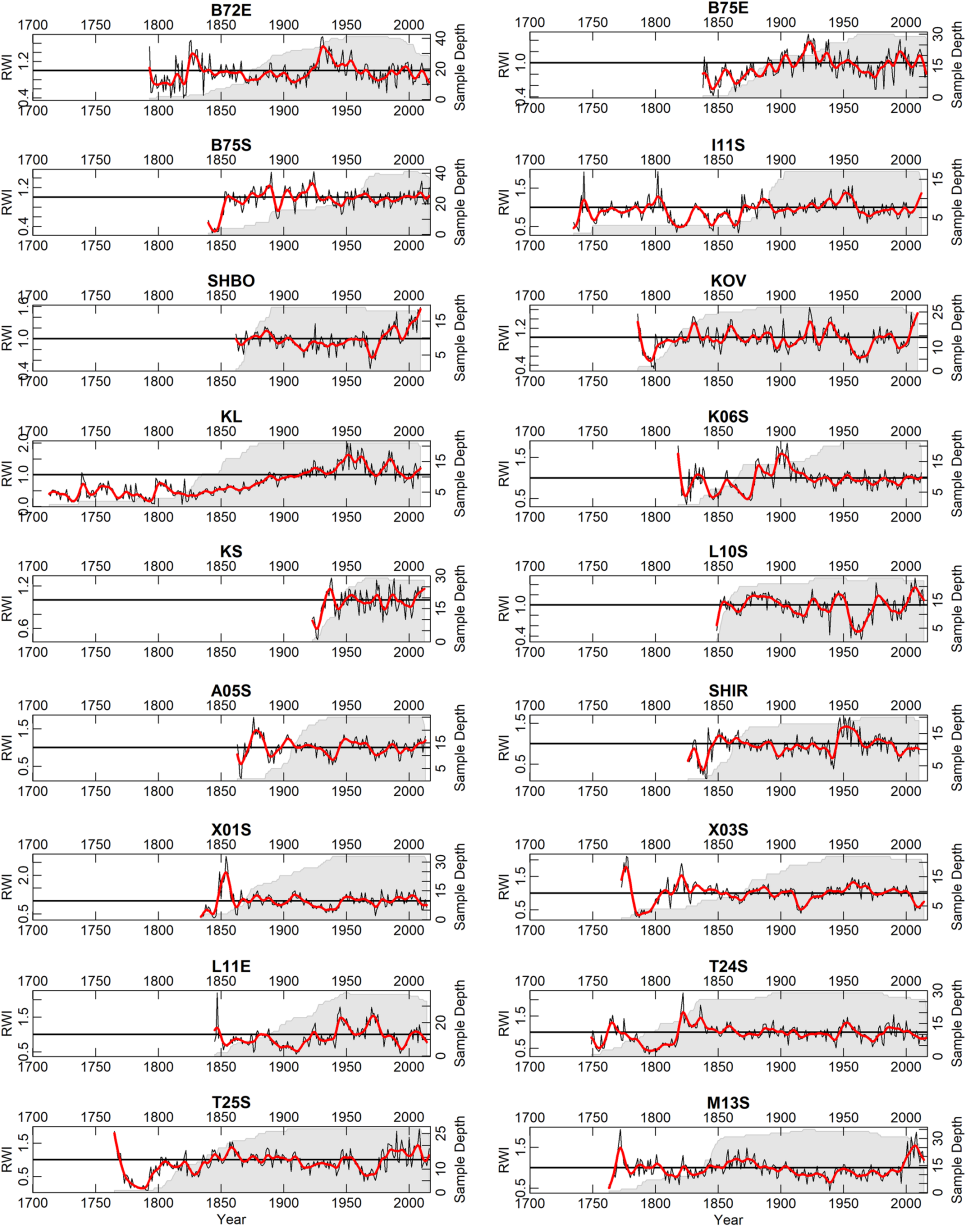

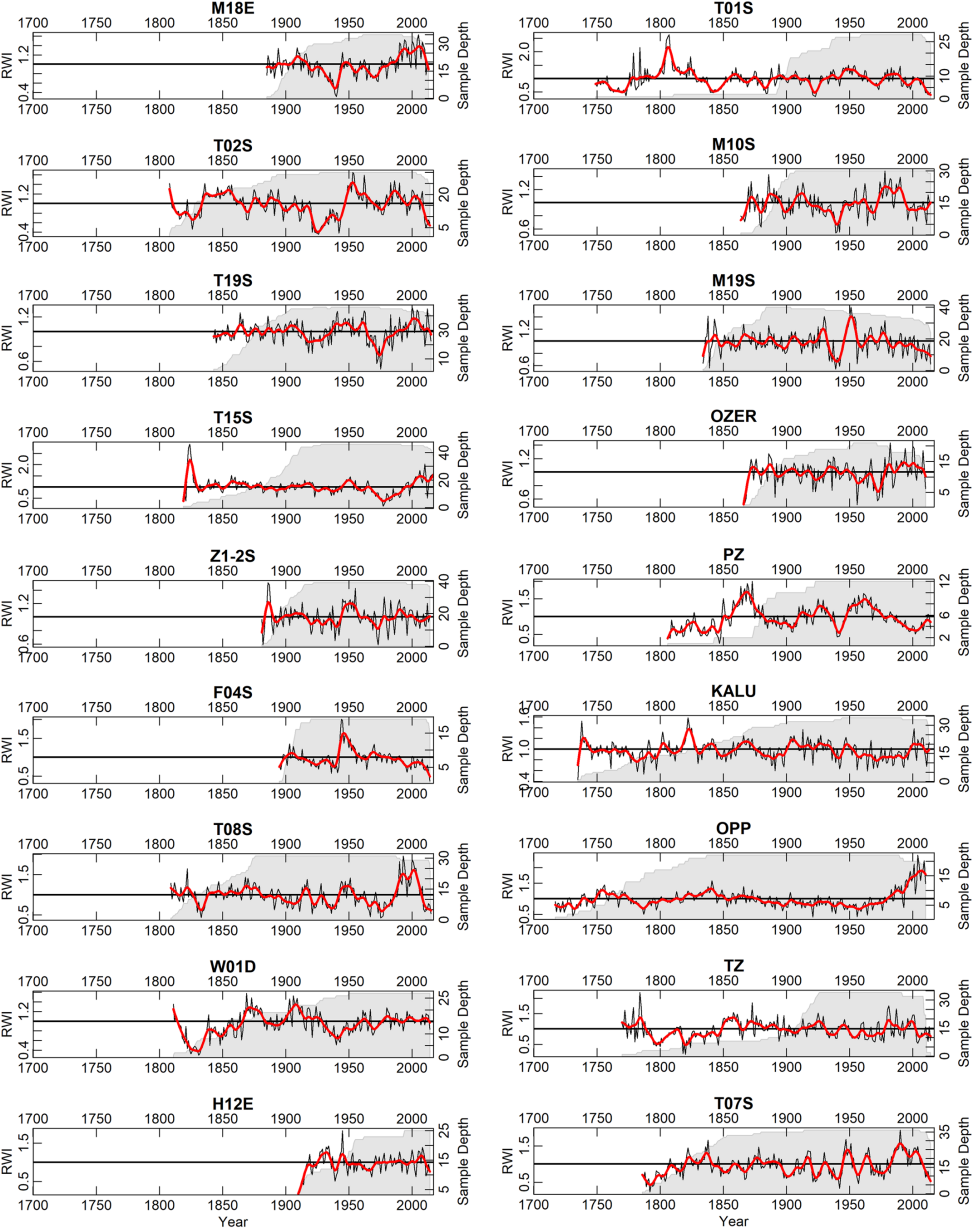

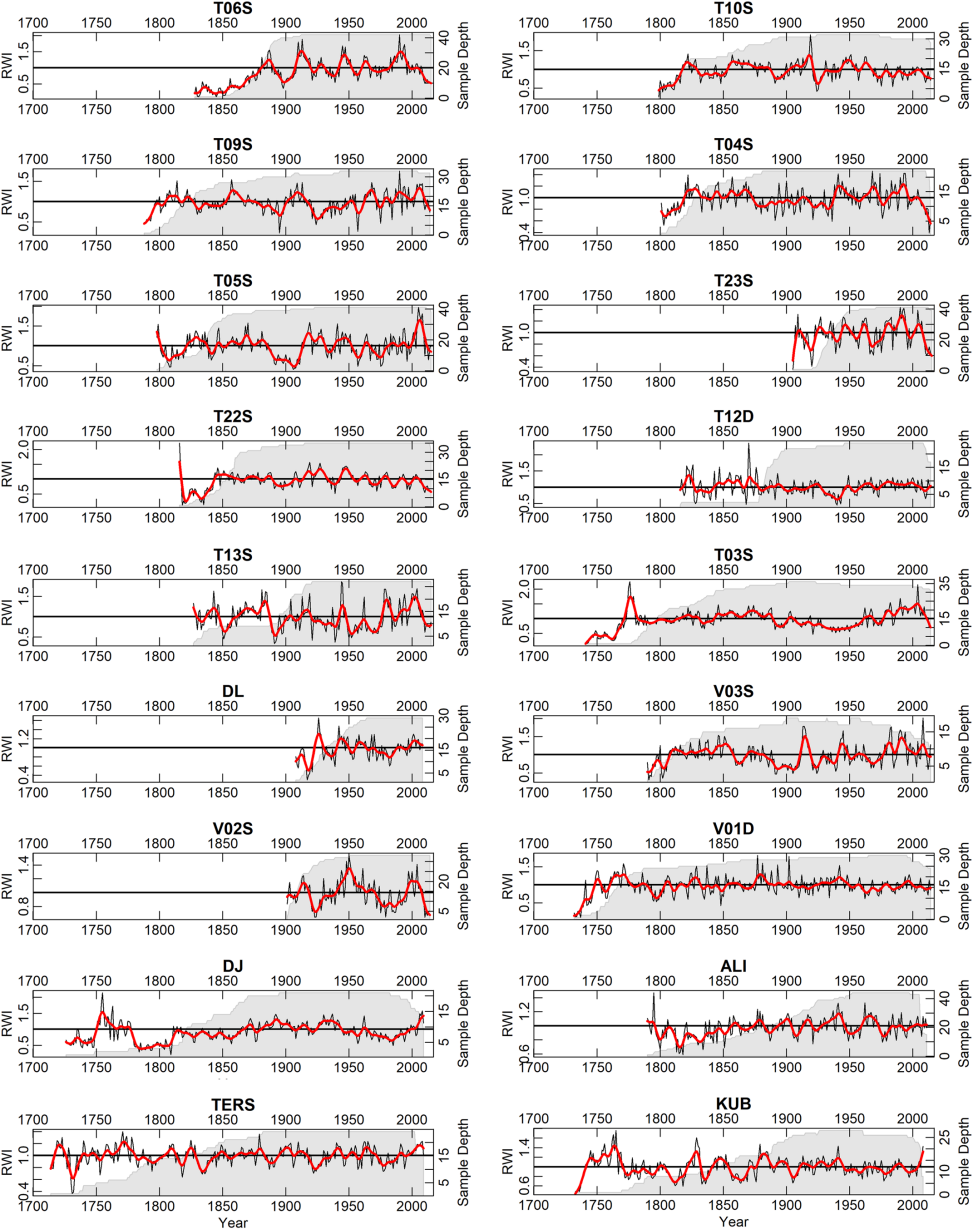
As Fig. 2, but for all the other ‘living-tree’ chronologies.

**Fig. 4:**
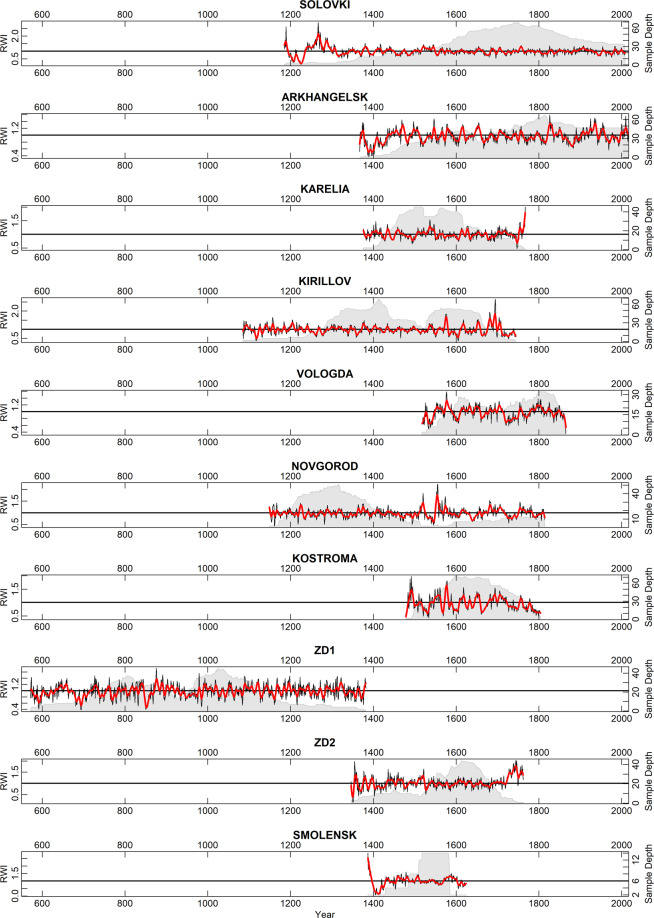
As Fig. 2, but for the ‘historical’ chronologies.

The presented data set closes a vast gap in the Eurasian dendrochronological network and will serve for various applications: climatic reconstructions of regional and hemispheric scale, dendrochronological dating of historical and cultural wood, ecological and remote sensing studies.

## Methods

### Region, climate and species

The study region includes the European Part of Russia (EPR) from its western boarders to the Ural Mountains in the East, from the Arctic coast to the Northern Caucasus in the South. The climate of the EPR is characterized by marked seasonal changes, with cold winters and warm summers. Most of the EPR, except for the southern part, is in the humid continental climate zone (by Köppen-Geiger classification). The summer isotherms are located sublatitudinally because summer temperature depends mainly on solar radiation. Mean July temperature is about + 8–10 °C at the northern part of the Kola Peninsula and about + 25 °C in the Caspian region. The temperature gradient is more pronounced from west to east than from south to north. The climate of the EPR is moderated by westerlies: Atlantic air masses provide increased temperatures and precipitation in winter, while summers are rather cooler and wetter due to westerly transport. The mean January temperature varies from −5 °C in the west to −20 °C in the east. The coldest part of the EPR is the north-east, with snow cover lasting up to 7 months. Total precipitation is about 600–800 mm/year between 55°N and 65°N. From the Arctic Circle to the north, snow cover gradually decreases to 350 mm. Also, total precipitation decreases from 55°N in a south-easterly direction. In the Lower Volga region, it is about 250–400 mm/year, and near the Caspian Sea it can be lower than 250 mm/year. In most of the EPR summer precipitation exceeds precipitation in winter.

The climate of the Caucasus is determined by its location at the junction of temperate and subtropic climate zones, as well as by its elevation. The Greater Caucasus Range (with summits above 5000 m a.s.l) serves as a barrier preventing humid Atlantic and Mediterranean air masses moving westward. Thus, total precipitation decreases from 3000 mm/year in the western and southern slopes, to 600–800 mm/year in the north-east. Temperature depends on elevation and slope exposition; on average southern slopes are 3 °C warmer than the northern slopes. The climate becomes more continental in the easterly direction, as the difference between summer and winter temperature increases, and the mean temperature at corresponding elevations increases from west to east.

The major biomes represented in the EPR are tundra, forest-tundra, forest, forest-steppe, semi desert and desert. The northern Kola Peninsula is covered by tundra and semi-tundra, the main tree species is dwarf birch (*Betula nana*). Boreal evergreen forests predominantly composed of pines are typical of the Fennoscandia region and the Northern Ridge area. All other territory to the north of 60°N is covered by boreal evergreen forests predominantly composed of spruce. The boundary between evergreen and mixed forest is 56–57°N. The mixed forest biome (the main species are oaks and pines) becomes narrower from east to west: it is located between 56–57°N and 52°N at the far west of the EPR, and between 56 and 58°N in the east. To the south of the mixed forests are the temperate broadleaf forests predominantly composed of oaks. The width of this zone is 200–300 km. The Caucasus plant communities vary with elevation. In the north-western part of the Caucasus mountains broadleaf forests are predominant at lower elevations, the dominant species between 400 and 1000 meters are Georgian oak (*Quercus iberica*) and European hornbeam (*Carpinus betulus*). Oriental beech (*Fagus orientalis*) and Caucasian oak (*Quercus macranthera*) are the predominant species between 1000- and 1500-meter elevation. The mixed forests above 1500-meter elevation include Caucasian spruce (*Picea orientalis*), Nordmann fir (*Abies nordmanniana*), and Caucasian pine (*Pinus sylvestris* var. hamata). Dwarf forests are above 1800 m.a.s.l., composed of oriental oak and Caucasian pine in drier areas, birch (*Betula pubescens* var. litwinowii and *Betula raddeana*) in more humid areas. In the Elbrus region, all altitudinal belts shift upwards because of increasing aridity and continentality^23^.

### Sampling and laboratory protocols

The aim of all the living tree sampling efforts was to produce long chronologies, primarily for use in dendroclimatic studies. First selection priority was given to old trees, both dominant and subdominant undisturbed individuals. Normally, two cores from the opposite sides of the tree were collected at breast height with an increment corer. At each site 10 to 22 trees were sampled, with some exceptions (online-only Table 1).

In the laboratory, the cores were dried, mounted and sanded (in some cases first flattened by a core microtome and then slightly sanded). Then the ring widths were either directly measured on LINTAB device or Velmex Tree Ring Measuring system or scanned with 2800–3600 dpi resolution and measured using CooRecorder software. In all cases, the measurement precision exceeded 0.01 mm.

Samples from wooden buildings and from wooden construction elements of stone buildings were usually collected by a specialized corer designed for use with an electric drill and produced by Schneidwerkzeugmechanik, Berlin (https://www.schneidwerkzeugmechanik.de/). In some cases, a traditional, manual increment corer was used, or cut from samples using a saw. Almost all archaeological wood was collected as saw-cuts. The cores from construction wood and the cores from living trees were treated in the laboratory equally. Wooden cuts were dried if wet, polished, and measured across two to four radii. In the case of poor preservation of the wooden samples, when it was impossible to achieve a reasonable quality of surface by polishing, the surface was prepared using a razor blade. For example, all the archaeological samples used in Kirillov chronology (online-only Table 2) were prepared this way.

## Data Records

The data set consists of tree-ring width measurements in Decadal/Tuscon RWL format^24^, COFECHA^25^ listings for every RWL file, online-only Tables 1 and 2 with the description for every living-tree and historical chronology. In each RWL file the measurements for each tree denoted by a number are usually represented by several cores denoted by the letters a,b,c, etc., e.g. T15S1a and T15S1b are two cores for the first tree at the site T15S, T15S15a and T15S15b are two cores for the 15^th^ tree at the site. The historical chronologies usually contain several codes referring to different sources of materials, but the numbering is the same – numbers denote different beams from each source and letters a-d denote the measurements along different radii from each beam.

Missing values in RWL files are denoted either by zeroes in the case of missing rings or by −888 in the case of missing core segments. The description of each site contains the information on the location, geographical coordinates, number of trees and samples, information on series intercorrelation, average mean sensitivity, quality of the cross-dating, and related publications (online-only Tables 1, 2). Some sites also have descriptions of vegetation and soils. The RWL files of the measurements and the related COFECHA quality control listings are publicly available in ITRDB. The ITRDB codes and links are provided in the online-only Tables 1 and 2. The whole data set is also available as a standalone set of files^26^ in Figshare repository, where RWL files are named as the site code plus ‘.rwl’ extension, the COFECHA listings are named as the site code plus ‘COF.txt’. For example, the site T15S is represented by the files ‘T15S.rwl’ and ‘T15SCOF.txt’. Supplementary Tables 1 and 2 represent printable versions of Online-only Tables 1 and 2, respectively.

Below we describe the sources of material for each historical chronology.

### Kirillov

Materials for the Kirillov chronology were collected over many years from archaeological excavations in the town of Kirillov, Vologda region. They include wood samples obtained from architectural buildings and various small archaeological excavations in the vicinity of the Kirillo-Belozersky monastery (59.86°N, 38.37°E). During restoration work in 1969, 1971, 1985, and 1987, samples of wooden ties and piles of foundations from brick defensive walls and monastery buildings were collected. The archaeological part of the collection also contains samples from wooden log cabins, wells, and log heaps (remnants of buildings demolished during renovation) and discovered during rescue excavations in 1994, 1998–2000, 2007, 2008, 2011, 2015, 2016, and 2018. The samples were processed in the Laboratory of Natural Science Methods in Archaeology, Institute of Archaeology RAS. Unfortunately, most of the original material has not been archived after the measurements were made. The Kirillov chronology was calendar dated with living trees from the Vologda region (sites KOV and SHBO) and materials from the Museum of Wooden Architecture of the Vologda Region “Semyonkovo”^27^.

### Vologda

The collection consists of materials from wooden buildings in the city of Vologda (59.22°N, 39.89°E). The data was assembled by D. Kats in the 1990s and later archived at the Institute of Plant and Animal Ecology in Ekaterinburg. In 2009 the collection was transferred again, and now resides at the Institute of Geography RAS, where ring-widths were measured a second time. The data set includes the samples from 19^th^ century wooden houses on Gogol Street, numbers 3 and 5 (codes AU and AV), from Gertsen Street number 58 (code BA), from the Spaso-Prilutskiy Monastery in the northern outskirts of Vologda (code BB), and from samples of unknown origin from the 18^th^ century (code M). The Vologda chronology was calendar dated with the Kirillov chronology.

### Novgorod

Materials in the Novgorod chronology are derived from archaeological excavations in the city of Velikiy Novgorod (58.52°N, 31.27°E), in addition to samples from wooden buildings of the Novgorod Region. The latter include materials from building transferred to the Museum of Wooden Architecture “Vitoslavlitsy” from the Novgorod region. These include the Chapel of Magdalena (code N04A), the Church of St. Nicolay from the village of Visokiy Ostrov (code N09A), and a church from the village of Tukholi (code N11A). Archaeological materials come from the city of Novgorod, from the excavation of Yaroslavovo Dvorische (archaeologist A.V. Andrienko, code N02A^28^), as well as excavations on Telegina-Redyatina Street (code ‘tere’), Posolskaya Street (code ‘posol’), Znamenskaya Street (code ‘znam’), Troitskaya Street (codes ‘35a-1-b1’ and ‘16a-1-v2’), and B. Konyushennaya Street (code ‘kon’), which were directed by archaeologist O.I. Oleynikov. The Novgorod chronology was calendar dated using the russ1 chronology from the ITRDB (with a correction for the known error of 1 year^29^), and by crossdating with the Kirillov and Vologda chronologies.

### Arkhangelsk

The Arkhangelsk chronology includes samples from houses and churches from the northwestern part of the Arkhangelsk region (63.4–64.7°N, 37.4–43.4°E). These include wooden houses from the town of Pinega, Kudrina Street 45 and 55 (codes I15A and I14A, 64.70°N, 43.39°E), the house of the Bazheniny family in the village of Vavchuga, Kholmogorskoye district (code I21A, 64.23°N, 41.92°E), the Church of Introduction in the village of Vorzogory (code I02A, 63.89°N, 37.67°E), the Church of Vladimir in the village of Medvedevskaya (code I04A, 63.81°N, 38.32°E), and from the the Ensemble of the Church in the village of Piyala (codes I08A, I09A, P, 63.43°N, 39.08°E), all located in the Onezhskiy District. The chronology was calendar dated using a living pine tree-ring series (code I24S, 64.11°N, 38.03°E) in addition to crossdating with the Solovki chronology^30^.

### Karelia

This chronology includes materials from eight churches in the Republic of Karelia, all located along the shores of Onega Lake (60.80–62.72°N, 33.06–35.27°E)^31^. Most of these measurements are of lower precision than of the other data in this study (0.05 mm versus 0.001 mm) however, they are vital to the dendrochronological dating in the region. The Karelia chronology was calendar dated using the Solovki and Arkhangelsk chronologies.

### Zapadnaya Dvina (ZD1, ZD2)

Tree-ring chronologies ZD1 and ZD2 were constructed with subfossil oak trees sampled in the alluvial deposits of the Zapadnaya Dvina River and its tributary, the Velesa River. The sample sites include reaches of both rivers upstream of their confluence (56.06°N, 31.97°E). Subfossil oak tree trunks were discovered in the riverbed as well as in riverbank alluvial deposits and oxbow lakes. The ZD1 and ZD2 chronologies do not overlap with the living oak tree-ring series from the region, but were crossdated with chronologies from Belarus and from the Baltic region. ZD1 (CE 572–1382) was calendar dated with oak samples from the Church of the Saviour’s Transfiguration in Polotsk (Belarus) which spans CE 869-1122^32^; it also crossdates with subfossil oak series from Smarhon, Belarus^33^ and the Baltic 1 chronology^34^. A detailed report was previously published elsewhere^14^. The calendar age of the ZD2 chronology (CE 1346–1762) was established by comparison with the 2021BLT3 chronology^35^.

### Kostroma

Materials for the Kostroma chronology come from archaeological excavations in the City of Kostroma and from the wooden buildings from the surrounding Kostroma Region. They include materials from a church in the Andreevskoye village (code K2A, 58.16°N, 41.30°E), two buildings from the Museum of Wooden Architecture in the Kostroma region, which include the house of Skobyolkin (code K13A), and the Church of Ilijah the Prophet (code K14A). The other materials come from the ‘Melochniye Ryady’ excavations in the center of Kostroma, (archaeologist A.Lazarev, code K09A). The chronology was calendar dated using the Kirillov and Vologda chronologies.

### Smolensk

Seven beams of pine come from archaeological excavations at Pobedy Square in the city of Smolensk (54.78°N, 32.05°E)^36^. They were crossdated using the chronology from the Dannenshtern House in Riga^37^. The material of the Dannenshtern House likely comes from near the headwaters of the Kasplya tributary of the Daugava River (Zapadnaya Dvina River) located near Smolensk.

### Solovki

The Solovki chronology consists of measurements from living trees (pines PDB and spruce PDEL; 65.12°N, 35.57°E), beams in a church on Malaya Muksalma Island (code MMCH; 65.01°N, 36.00°E), a building built for resin extraction (code SMOL), a barn (code SOLAM), and from a monastery outbuilding (or skit) on Sekirnaya Hill (code SLKL; 64.08°N, 35.57°E). Also included in the chronology are series from a satellite monastery building on Bolshaya Muksalma Island (code BMSK; 65.03°N, 35.90°E), series from a bathhouse nearby (code BMBN), samples from the Church of Andrew the First-Called on Zayatskiy Island (code B24A; 64.97°N, 35.65°E), series from a 19^th^ century building (code SOLIZ), along with archaeological materials from the monastery (codes B27A, B28A), and a barn on Anzer Island (codes B39A, B38A; 65.19°N, 35.98°E). The earliest part of the chronology consists of ring-width series from beams from the 16^th^ century Spaso-Preobrazhenskiy Cathedral (code SP; 65.02°N, 35.71°E).

## Technical Validation

Measurements were validated statistically with program COFECHA^25^. COFECHA separates each series of tree-ring measurements into 50-yr segments with 25-yr overlap and calculates correlation of each segment (its high-frequency band) with the average high frequency value of all the other series of a file for the same 50-yr segment. The independent comparison of each segment helps to identify possible problems of measurement quality and cross-dating in different parts of the series. Some segments may be marked by ‘A’ or ‘B’ flag. An ‘A’ flag marks an insignificant correlation (*r < 0.328*). A ‘B’ flag marks segments that aligned with the master series with higher correlation coefficient in another position within + /− 10 years. For every site the percentage of segments with ‘A’ and ‘B’ flags was calculated to describe the overall quality of measurements and their cross-dating. Additional validation included alignment of every chronology with other chronologies of the same region and species to make sure there are no systematic dating errors.

## Supplementary information

Supplementary information

## Data Availability

No code was used to generate, test, or process the current dataset.

## Online-only Tables

**Online-only Table 1:** Description of ‘living-tree’ chronologies. The chronologies are represented from North to South.

Site code	Location	Description of the site	Latitude, degrees N	Longitude, degress E	Height, m a.s.l.	Species	No. of trees	No. of cores	Investigatiors	First year	Last year	Publications (described in,first used in)	ITRDB code	ITRDB link	Mean series intercorrelation (COFECHA)	Average mean sensitivity (COFECHA)	COFECHA quality (A-very good, B - good, C - moderate)*	No. of segments in COFECHA	Possible problems	% of segments with possible problems	Additional notes
B36S	Arkhangelsk region, Primorskiy district, Solovetskiye islands	Anzer island, mixed forest on the way to Troitskiy skit	65.18	35.97	10	PISY	17	27	Solomina O.N., Dolgova E.A.	1549	2010	^38^	RUS318	https://www.ncei.noaa.gov/access/paleo-search/study/36316	0.58	0.2	B	330	18	5.5	
B42S	Arkhangelsk region, Primorskiy district, Solovetskiye islands	pine forest near cape Rebolda and Bannoye lake	65.15	35.8	10	PISY	12	23	Solomina O.N., Dolgova E.A.	1537	2011	^38^	RUS319	https://www.ncei.noaa.gov/access/paleo-search/study/36317	0.577	0.205	A	370	3	0.8	
B72E	Arkhangelsk region, Primorskiy district, Solovetskiye islands	near eastern part of the Krasnoye lake, 10–20 degree slope, spruce forest with aspen (green moss, firn, blueberry)	65.09063	35.66825	20	PCAB	21	42	Solomina O.N., Dolgova E.A.	1789	2016	not published	RUS320	https://www.ncei.noaa.gov/access/paleo-search/study/36318	0.662	0.223	A	210	5	2.4	
B75S	Arkhangelsk region, Primorskiy district, Solovetskiye islands	on the way to cape Tolstik (young and middle-aged pines)	65.03915	35.64086	9	PISY	21	42	Solomina O.N., Mikhalenko V.N., Dolgova E.A.	1840	2016	^38^	RUS321	https://www.ncei.noaa.gov/access/paleo-search/study/36319	0.613	0.238	A	131	6	4.6	
B75E	Arkhangelsk region, Primorskiy district, Solovetskiye islands	on the way to cape Tolstik (young and middle-aged spruces)	65.03915	35.64086	9	PCAB	20	30	Solomina O.N., Mikhalenko V.N., Dolgova E.A.	1838	2016	not published	RUS322	https://www.ncei.noaa.gov/access/paleo-search/study/36320	0.598	0.185	A	138	4	2.9	
I12L	Arkhangelsk region, Pinega district	Pinega preserve	64.6948333	43.1884444	29	LASI	17	31	Solomina O.N., Matskovsky V.V., Dolgikh A.V.	1670	2012	^19^^,^^22^	RUS332	https://www.ncei.noaa.gov/access/paleo-search/study/36330	0.53	0.248	A	231	8	3.5	
I11S	Arkhangelsk region, Pinega district	Pinega preserve	64.5631389	43.1615556	90	PISY	10	17	Solomina O.N., Matskovsky V.V., Dolgikh A.V.	1735	2012	^19^^,^^22^	RUS331	https://www.ncei.noaa.gov/access/paleo-search/study/36329	0.506	0.205	B	99	9	9.1	
SHBO	Vologda region, Kirillov district, Shalgo-Boduny forestry	pine forest, trees with scars from resin collection	60.45	38.46	171	PISY	10	19	Solomina O.N., Matskovsky V.V.	1862	2009	^19^^,^^22^^,^^27^	RUS354	https://www.ncei.noaa.gov/access/paleo-search/study/36352	0.566	0.196	A	103	2	1.9	trees with scars from resin collection
KOV	Vologda region, Kirillov district, Kovarzino forestry	pine forest on a bog	60.16	38.84	138	PISY	15	27	Solomina O.N., Matskovsky V.V.	1786	2009	^19^^,^^22^^,^^27^	RUS340	https://www.ncei.noaa.gov/access/paleo-search/study/36338	0.556	0.207	A	204	0	0.0	
KL	Kologriv, Kostroma region, Kologriv district	Kologriv forest preserve	58.982	44.379	200	PCAB	10	21	Solomina O.N.	1713	2009	^19^^,^^22^	RUS338	https://www.ncei.noaa.gov/access/paleo-search/study/36336	0.548	0.258	A	149	4	2.7	
K06S	Kologriv, Kostroma region, Kologriv district, near town Kologriv		58.8661	44.2336	141	PISY	12	21	Solomina O.N.	1836	2012	^19^^,^^22^	RUS333	https://www.ncei.noaa.gov/access/paleo-search/study/36331	0.475	0.226	B	114	9	7.9	
KS	Kostroma region, Manturocskiy district	near town Manturovo, floodplain decidous forest with oaks	58.1505	44.4952	100	QURO	17	30	Khasanov B.F.	1923	2012	^39^	RUS341	https://www.ncei.noaa.gov/access/paleo-search/study/36339	0.533	0.227	B	77	5	6.5	
L10S	Tver region, Rameshkovsky district, near Kiverichi village		57.345825	36.618622	155	PISY	12	24	Solomina O.N.	1849	2014	^19^^,^^22^	RUS344	https://www.ncei.noaa.gov/access/paleo-search/study/36342	0.552	0.242	B	130	7	5.4	
A05S	Yaroslavl region, Rostov district, Borisoglebsky town	pine forest	57.2524	39.1637	138	PISY	13	25	Solomina O.N., Matskovsky V.V.	1863	2013	^19^^,^^22^	RUS315	https://www.ncei.noaa.gov/access/paleo-search/study/36313	0.531	0.232	A	103	1	1.0	
SHIR	Tver region, Penovskiy district, near Shirkovo village		57.0491	32.733	224	PISY	13	20	Solomina O.N.	1826	2010	^19^^,^^22^	RUS355	https://www.ncei.noaa.gov/access/paleo-search/study/36353	0.593	0.25	A	113	4	3.5	
X01S	Nizhniy Novgorod region, Zelyoniy gorod	Child summer camp named after Talalashkin	56.54353	44.79688	108	PISY	18	33	Solomina O.N.	1834	2014	^19^^,^^22^	RUS383	https://www.ncei.noaa.gov/access/paleo-search/study/36381	0.595	0.246	A	126	0	0.0	
X03S	Nizhniy Novgorod region, Semyonovskiy district	Kerzhensky Nature Reserve, 46 block, pine forest with spruce, lingonberry and green moss	56.54353	44.79688	108	PISY	12	23	Solomina O.N.	1773	2014	^19^^,^^22^	RUS384	https://www.ncei.noaa.gov/access/paleo-search/study/36382	0.5	0.2	A	253	11	4.3	
L11E	Tver region, Nelidovo district	Central forest preserve	56.458	32.958888	244	PCAB	19	38	Solomina O.N., Matskovsky V.V., Dolgova E.A.	1845	2014	^19^^,^^22^	RUS345	https://www.ncei.noaa.gov/access/paleo-search/study/36343	0.573	0.253	A	168	3	1.8	
Y01S	Republik of Mariy El, Volzhskiy district	National Park “Mariy Chodra”	56.0493611	48.4616944	87	PISY	24	44	Solomina O.N., Matskovsky V.V., Kuznetsova V.V., Tishin D.V.	1802	2014	^19^^,^^22^^,^^40^	RUS385	https://www.ncei.noaa.gov/access/paleo-search/study/36383	0.547	0.222	A	168	2	1.2	
T24S	Republic of Tatarstan, Zelenodolskiy district	Volzhsko-Kamsky reserve, Raifskoe forestry, quarter 25. Forest 200 m from the edge	55.90901	48.73328	86	PISY	15	29	Solomina O.N., Kuznetsova V.V.	1749	2016	^19^^,^^41^^,^^42^	RUS374	https://www.ncei.noaa.gov/access/paleo-search/study/36372	0.601	0.234	A	230	5	2.2	
T25S	Republic of Tatarstan, Zelenodolskiy district	Volzhsko-Kamsky reserve, Raifskoe forestry, quarter 25. Edge of the forest	55.90783	48.73229	79	PISY	15	27	Solomina O.N., Kuznetsova V.V.	1765	2016	^19^^,^^22^^,^^40^^,^^42^	RUS375	https://www.ncei.noaa.gov/access/paleo-search/study/36373	0.632	0.273	A	190	3	1.6	
M13S	Moscow region, Zvenigorod town	pines around the ancient church	55.733765	36.840801	167	PISY	20	35	Solomina O.N., Matskovsky V.V.	1763	2014	^19^^,^^22^	RUS347	https://www.ncei.noaa.gov/access/paleo-search/study/36345	0.49	0.245	B	243	18	7.4	
M18E	Moscow region, Zvenigorod district, Zvenigorod biological station		55.696133	36.727998	199	PCAB	18	35	Solomina O.N., Matskovsky V.V.	1885	2013	^19^^,^^22^	RUS348	https://www.ncei.noaa.gov/access/paleo-search/study/36346	0.56	0.266	A	144	6	4.2	
T01S	Republic of Tatarstan, Laishevskiy district, near Saraly village	Volga-Kama natural reserve. Saralovsky site, forest-steppe, trees grow under a dune in a green moss forest of a lowland	55.3	49.26	66	PISY	15	25	Solomina O.N., Matskovsky V.V., Kuznetsova V.V., Tishin D.V.	1714	2014	^19^^,^^22^^,^^40^^,^^42^	RUS358	https://www.ncei.noaa.gov/access/paleo-search/study/36356	0.591	0.271	A	120	3	2.5	
T02 S	Republic of Tatarstan, Laishevskiy district, near Saraly village	Volga-Kama natural reserve. Saralovsky site, forest-steppe, trees grow on dunes in a lichen forest	55.3	49.26	75	PISY	16	30	Solomina O.N., Matskovsky V.V., Kuznetsova V.V., Tishin D.V.	1808	2014	^19^^,^^22^^,^^40^^,^^42^	RUS359	https://www.ncei.noaa.gov/access/paleo-search/study/36357	0.583	0.244	A	204	6	2.9	
M10S	Ryazan region, Spas-Klepiki district, Mesherskiy bor	pine forest	55.2372	40.0368	125	PISY	15	30	Solomina O.N.	1864	2014	^19^^,^^22^	RUS346	https://www.ncei.noaa.gov/access/paleo-search/study/36344	0.5	0.231	A	148	2	1.4	
T19S	Republic of Chuvashia, Alatyrskiy district	Prisurskiy preserve, temperate forest, highland	55.04159	46.59458	87	PISY	22	47	Solomina O.N., Kuznetsova V.V.	1843	2015	^19^^,^^22^^,^^40^^,^^42^	RUS371	https://www.ncei.noaa.gov/access/paleo-search/study/36369	0.548	0.203	B	252	16	6.3	
M19S	Moscow region, Serpukhov district	Prioksko-terrasny preserve	54.9123	37.6564	181	PISY	25	40	Solomina O.N., Dolgova E.A.	1834	2014	^19^^,^^22^	RUS349	https://www.ncei.noaa.gov/access/paleo-search/study/36347	0.596	0.23	A	231	4	1.7	
T15S	Republic of Chuvashiya, Shemurshinskiy district	National Park “Chavash Varmane”	54.9	47.51	200	PISY	28	46	Solomina O.N., Kuznetsova V.V.	1819	2017	Not published	RUS370	https://www.ncei.noaa.gov/access/paleo-search/study/36368	0.621	0.303	A	232	7	3.0	
OZER	Moscow region, Kolomenskiy district, near Ozyory town		54.857721	38.548875	134	PISY	14	21	Solomina O.N.	1866	2010	^19^^,^^22^	RUS352	https://www.ncei.noaa.gov/access/paleo-search/study/36350	0.575	0.244	A	96	1	1.0	
Z1–2S	Republik of Mordoviya, Temnikovskiy district	Mordovian Nature Reserve, 396 block, spruce-pine forest, grassy, with young oak	54.770816	43.4048833	180	PISY	21	39	Solomina O.N.	1881	2014	^19^^,^^22^	RUS386	https://www.ncei.noaa.gov/access/paleo-search/study/36384	0.511	0.229	A	179	7	3.9	
PZ	Kaluga region, Dzerzhinskiy district	town Polotnyany Zavod, park with pines and oaks	54.7323	36.0002	170	QURO	7	12	Khasanov B.F.	1806	2014	^43^	RUS353	https://www.ncei.noaa.gov/access/paleo-search/study/36351	0.572	0.22	C	63	7	11.1	
F04S	Smolensk region, Ugransky distrikt, Ugra village		54.66108	34.13667	195	PISY	11	19	Solomina O.N.	1895	2014	^19^^,^^22^	RUS329	https://www.ncei.noaa.gov/access/paleo-search/study/36327	0.559	0.226	A	80	2	2.5	
KALU	Kaluga region, Kaluga town	pine forest	54.529614	36.200908	183	PISY	18	34	Solomina O.N., Matskovsky V.V., Dolgova E.A., Kuznetsova V.V.,	1735	2013	^19^^,^^22^	RUS334	https://www.ncei.noaa.gov/access/paleo-search/study/36332	0.503	0.253	C	258	27	10.5	
T08S	Tatarstan, Bugulminsky district	Linden-oak forest stands on typical, thin leached and carbonate chernozems. The dendrochronological site was established on the slope of the southern exposure, not far from the village of Petrovka. The hilly territory is dissected by a dense river and gully (the depth of the erosional incision is up to 120 m). Due to the microclimatic features, forest stands may be affected by frosts in June and August.	54.426754	52.757354	240	PISY	18	31	Solomina O.N., Matskovsky V.V., Kuznetsova V.V., Tishin D.V.	1809	2015	^19^^,^^22^^,^^40^^,^^42^	RUS365	https://www.ncei.noaa.gov/access/paleo-search/study/36363	0.627	0.269	A	210	2	1.0	
OPP	Kaluga region, Kozelsk district, Optina pustin’ monastery	pine forest near monastery	54.049325	35.830665	154	PISY	12	22	Solomina O.N., Matskovsky V.V., Dolgova E.A.	1717	2010	^19^^,^^22^	RUS351	https://www.ncei.noaa.gov/access/paleo-search/study/36349	0.526	0.264	C	208	24	11.5	
W01D	Tula region, Schekinskiy disctrict	Arboretum of Krapivensk Forestry College (Selivanovo village)	53.98837	37.25537	175	QURO	15	27	Solomina O.N.	1809	2014	^19^^,^^22^	RUS382	https://www.ncei.noaa.gov/access/paleo-search/study/36380	0.553	0.218	B	151	13	8.6	
TZ	Tula region, Schekinskiy disctrict	near town Krapivna, decidous broadleaf forest with limes, maples, oaks and ashes	53.9781	37.1162	180	QURO	16	34	Khasanov B.F.	1770	2014	^43^	RUS377	https://www.ncei.noaa.gov/access/paleo-search/study/36375	0.652	0.287	A	169	2	1.2	
H12E	Kaluga region, Kozelsk, near town Kozelsk		53.9633	35.8131	166	PCAB	13	25	Solomina O.N., Matskovsky V.V., Dolgova E.A., Kuznetsova V.V.,	1910	2014	^19^^,^^22^	RUS330	https://www.ncei.noaa.gov/access/paleo-search/study/36328	0.585	0.289	A	75	2	2.7	
T07S	Samara region, Stavropolskiy district	Ziguly national reserve, broadleaf-pine and pine forests on carbonate and gray soils. Due to the high fracturing of limestone rocks on the territory of the reserve there are no permanent streams. The cite locates on the western slope of Strelnaya hill	53.44	49.78	342	PISY	20	36	Solomina O.N., Matskovsky V.V., Kuznetsova V.V., Tishin D.V.	1786	2014	^19^^,^^22^^,^^40^^,^^42^	RUS364	https://www.ncei.noaa.gov/access/paleo-search/study/36362	0.591	0.296	A	270	8	3.0	
T06S	Samara region, Stavropolskiy district	Ziguly national reserve, broadleaf-pine and pine forests on carbonate and gray soils. Due to the high fracturing of limestone rocks on the territory of the reserve there are no permanent streams. The cite locates on the slope of a gully	53.406382	49.973071	162	PISY	21	42	Solomina O.N., Matskovsky V.V., Kuznetsova V.V., Tishin D.V.	1828	2015	^19^^,^^22^^,^^40^^,^^42^	RUS363	https://www.ncei.noaa.gov/access/paleo-search/study/36361	0.705	0.277	A	221	0	0.0	
T10S	Penza region, Kuznetskiy district	“Privolzhskaya lesostep” nature reserve	53.36332	46.89575	292	PISY	18	32	Solomina O.N., Kuznetsova V.V.	1799	2014	^19^^,^^22^^,^^40^^,^^42^	RUS367	https://www.ncei.noaa.gov/access/paleo-search/study/36365	0.565	0.237	B	223	12	5.4	
T09S	Penza region, Kuznetskiy district	“Privolzhskaya lesostep” nature reserve	53.32105	46.89051	257	PISY	20	34	Solomina O.N., Kuznetsova V.V.	1788	2014	^19^^,^^22^^,^^40^^,^^42^	RUS366	https://www.ncei.noaa.gov/access/paleo-search/study/36364	0.645	0.281	A	236	7	3.0	the trees were used for resin collection
T04S	Orenburg region, Buzulukskiy district	National Park “Buzulukskiy Bor”, Buzuluk pine forest	53.03	52.11	83	PISY	11	22	Solomina O.N., Matskovsky V.V., Kuznetsova V.V., Tishin D.V.	1801	2014	^19^^,^^22^^,^^40^^,^^42^	RUS361	https://www.ncei.noaa.gov/access/paleo-search/study/36359	0.577	0.232	A	159	7	4.4	
T05S	Orenburg region, Buzulukskiy district	National Park “Buzulukskiy Bor”, Buzuluk pine forest	52.95386	52.05854	79	PISY	21	41	Solomina O.N., Matskovsky V.V., Kuznetsova V.V., Tishin D.V.	1798	2015	^19^^,^^22^^,^^40^^,^^42^	RUS362	https://www.ncei.noaa.gov/access/paleo-search/study/36360	0.675	0.271	A	282	6	2.1	
T23S	Saratov Region, Khvalynskiy district	Khvalynsky National Park, is located in the southeast of the Volga Upland, a territory dissected by a dense net of gullies dominated by skeletal soils of the chernozem type. Site T23S was established in a pine forest on a flat area near the village. Elshanka	52.58	48	256	PISY	21	41	Solomina O.N., Kuznetsova V.V.	1905	2015	^19^^,^^40^^,^^42^	RUS373	https://www.ncei.noaa.gov/access/paleo-search/study/36371	0.669	0.236	A	122	0	0.0	
T22S	Saratov Region, Khvalynskiy district	Khvalynsky National Park, is located in the southeast of the Volga Upland, a territory dissected by a dense net of gullies dominated by skeletal soils of the chernozem type. Site T23S was established in a pine forest on a flat area near the village. Elshanka	52.5	48.04	270	PISY	19	35	Solomina O.N., Kuznetsova V.V.	1815	2015	^19^^,^^22^^,^^40^^,^^42^	RUS372	https://www.ncei.noaa.gov/access/paleo-search/study/36370	0.591	0.244	A	221	0	0.0	
T12D	Lipetsk region, Gryazenskiy district	near Faschovka village	52.46	39.69	136	QURO	12	24	Solomina O.N., Mikhalenko V.N.	1816	2014	^19^	RUS368	https://www.ncei.noaa.gov/access/paleo-search/study/36366	0.615	0.23	A	123	0	0.0	
T13S	Tambov region, Inzhavinskiy district	Karay-Saltykovo estate	52.36	42.6	128	PISY	15	27	Solomina O.N., Mikhalenko V.N.	1827	2015	^19^^,^^22^	RUS369	https://www.ncei.noaa.gov/access/paleo-search/study/36367	0.667	0.308	A	141	1	0.7	
T03S	Voronezh region, Bobrovsky district, Khrenovskoy forest	The southernmost natural pine forest in European Russia. Morozovskaya grove	51.201134	40.19928	94	PISY	21	36	Solomina O.N., Matskovsky V.V., Matveev S.M.	1741	2014	^12^^,^^19^^,^^21^^,^^22^^,^^44^	RUS360	https://www.ncei.noaa.gov/access/paleo-search/study/36358	0.557	0.234	A	276	7	2.5	
DL	Saratov region, Krasnokutskiy district	near town Krasny Kut, patches of decidous broadleaf oak forests amongst steppe	50.7299	46.6695	60	QURO	16	29	Khasanov B.F.	1908	2008	Not published	RUS328	https://www.ncei.noaa.gov/access/paleo-search/study/36326	0.58	0.255	A	89	1	1.1	
V03S	Belgorod region, Novooskolskiy district	Belogorie preserve, “Stenki izgoriya” part, chalk subsrate	50.6833	37.795988	100	PISY	14	22	Solomina O.N., Matskovsky V.V.	1790	2014	^19^^,^^22^	RUS380	https://www.ncei.noaa.gov/access/paleo-search/study/36378	0.576	0.282	A	131	6	4.6	
V02S	Belgorod region, Borisovskiy district	Belogorie preserve, “Les na Vorskle” part	50.6164167	35.9386388	148	PISY	17	33	Solomina O.N., Matskovsky V.V.	1901	2014	^19^^,^^22^	RUS379	https://www.ncei.noaa.gov/access/paleo-search/study/36377	0.501	0.223	B	126	12	9.5	
V01D	Belgorod region, Borisovskiy district	Belogorie preserve, “Les na Vorskle” part	50.604055	35.981444	165	QURO	16	30	Solomina O.N., Matskovsky V.V.	1732	2014	^19^^,^^22^	RUS378	https://www.ncei.noaa.gov/access/paleo-search/study/36376	0.758	0.234	A	268	1	0.4	
DJ	Krasnodar region, Mostovskiy district	Jubga Ridge	43.8820361	40.4678389	2100	PISY	13	21	Grabenko E.A, Kuderina T.M., Kudikov A.V.	1726	2009	Not published	RUS327	https://www.ncei.noaa.gov/access/paleo-search/study/36325	0.477	0.234	B	151	15	9.9	
D03F	Krasnodarskiy region, Sochi district, Krasnaya Polyana	Khmelevskie lakes, beech forest on the steep slope with North-East orientation	43.7186306	40.2050833	1806	FAOR	11	20	Solomina O.N., Dolgova E.A.	1680	2011	Not published	RUS325	https://www.ncei.noaa.gov/access/paleo-search/study/36323	0.657	0.377	A	180	7	3.9	
KHTP	Republic of Karachayevo-Cherkessiya, Karachaevskiy district	Teberda State Natural Biosphere Reserve, left and right sides of Malaya Khatipara Valley, upper tree limit	43.43025	41.70855	2285	PISY	38	70	Solomina O.N., Dolgova E.A.	1678	2010	^17^	RUS336	https://www.ncei.noaa.gov/access/paleo-search/study/36334	0.558	0.191	A	507	15	3.0	
KYZ	Republic of Karachayevo-Cherkessiya, Karachaevskiy district	upper-tree limit of Kyzgych valley	43.428	41.3035333	2392	PISY	20	12	Solomina O.N., Dolgova E.A.	1550	2007	^17^	RUS343	https://www.ncei.noaa.gov/access/paleo-search/study/36341	0.625	0.206	A	159	0	0.0	
CHM	Republic of Karachayevo-Cherkessiya, Karachaevskiy district	Teberda State Natural Biosphere Reserve, left side of the Kyzgych Valley	43.4043167	41.3109667	1714	PCAB	16	28	Solomina O.N., Dolgova E.A., Mikhalenko V.N.	1466	2011	^17^	RUS323	https://www.ncei.noaa.gov/access/paleo-search/study/36321	0.623	0.145	A	346	12	3.5	
ALI	Republic of Karachayevo-Cherkessiya, Karachaevskiy district	Teberda State Natural Biosphere Reserve, forefield of the Alibek Glacier	43.298383	41.57085	1889	PCAB	32	45	Solomina O.N., Dolgova E.A., Mikhalenko V.N.	1790	2011	^17^^,^^45^	RUS316	https://www.ncei.noaa.gov/access/paleo-search/study/36314	0.53	0.168	A	229	6	2.6	trees were sampled on the moraines of Alibek glacier
CHS	Republic of Kabardino-Balkariya, Elbrus district	Elbrus National Park, upper reaches of the Baksan river, Cheget Ridge, pine forest between avalanche tranzitional zones	43.260768	42.518934	2470	PISY	10	18	Solomina O.N., Dolgova E.A.	1694	2011	^17^	RUS324	https://www.ncei.noaa.gov/access/paleo-search/study/36322	0.484	0.139	C	151	20	13.2	
TERS	Republic of Kabardino-Balkariya, Elbrus district	Elbrus National Park, Terskol Glacier forefield	43.238	42.5056	2470	PISY	14	24	Solomina O.N., Dolgova E.A.	1714	2009	^17^^,^^46^	RUS376	https://www.ncei.noaa.gov/access/paleo-search/study/36374	0.525	0.163	A	200	3	1.5	
KUB	Republic of North Ossetia, Irafskiy district	slope of the Kubus Mt	42.8944	43.5906667	2356	PISY	18	29	Solomina O.N., Dolgova E.A., Matskovsky V.V.	1733	2008	^17^	RUS342	https://www.ncei.noaa.gov/access/paleo-search/study/36340	0.513	0.185	A	178	7	3.9	
D61S	Republic of North Osetia, Irafskiy district	the right slope of the Karaugom Glacier Valley	42.8548	43.708	2015	PISY	21	38	Solomina O.N., Dolgova E.A.	1600	2014	Not published	RUS326	https://www.ncei.noaa.gov/access/paleo-search/study/36324	0.602	0.219	A	355	14	3.9	

* COFECHA quality: A - less than 5% of segments with possible problems, B - from 5% to 10% of segments with possible problems, C - more 10% of segments with possible problems.

**Online-only Table 2:** Description of ‘historical’ chronologies. The chronologies are represented from North to South.

Site code	Location	Description of the site	Latitude, Southernmost, degrees N	Latitude, Northernmost, degrees N	Longitude, Westernmost, degrees E	Longitude, Easternmost, degrees E	Height, m a.s.l.	Species	No. of trees	No. of cores	Investigatiors	First year	Last year	Publications (described in, first used in)	ITRDB code	ITRDB link	Mean series intercorrelation (COFECHA)	Average mean sensitivity (COFECHA)	COFECHA quality (A-very good, B - good, C - moderate)*	No. of segments in COFECHA	Possible problems	% of segments with possible problems	Additional notes
SOLOVKI	Arkhangelsk region, Solovetskiy archipelago	wooden houses and churches from Solovetskiy archipelago, living trees	64.95	65.19	35.64	36.01	—	PISY, PCAB	75	112	Solomina O.N., Dolgova E.A., Matskovsky V.V., Semenyak N.S., Mikhalenko V.N.	1185	2008	^30^^,^^47^	RUS357	https://www.ncei.noaa.gov/access/paleo-search/study/36355	0.545	0.225	B	778	70	9.0	
ARKHANGELSK	Arkhangelsk region	wooden houses and churches from Arkhangelsk region	63.4	64.7	37.4	43.4	—	PISY, PCAB	45	90	Solomina O.N., Matskovsky V.V., Semenyak N.S.	1367	2020	^48^ [partly published]	RUS317	https://www.ncei.noaa.gov/access/paleo-search/study/36315	0.492	0.209	B	926	78	8.4	
KARELIA	Republic of Karelia, aroung Onega Lake	wooden churches	60.8	62.72	33.06	35.27	—	PISY, PCAB	67	34	Karpukhin A.A., Solov’yeva L.N., Chernykh N.B.	1376	1767	^31^	RUS335	https://www.ncei.noaa.gov/access/paleo-search/study/36333	0.415	0.195	C	463	72	15.6	
KIRILLOV	Vologda region, Kirillov district, Kirillov town	archaeological materials from Kirillo-Belozersky monastery and the city of Kirillov	59.86	59.86	38.37	38.37	—	PISY, PCAB	71	138	Karpukhin A.A., Solov’yeva L.N., Matskovsky V.V., Solomina O.N.	1085	1744	^22^^,^^27^	RUS337	https://www.ncei.noaa.gov/access/paleo-search/study/36335	0.511	0.214	C	946	135	14.3	
VOLOGDA	Vologda region, Vologda city	wooden houses and churches from the city of Vologda	59.22	59.22	39.89	39.89	—	PISY, PCAB	105	206	Solomina O.N., Matskovsky V.V	1518	1881	Not published	RUS381	https://www.ncei.noaa.gov/access/paleo-search/study/36379	0.507	0.206	C	357	101	28.3	
NOVGOROD	Novgorod region, Novgorod city	archaeological materials from the city of Novgorod	58.52	58.52	31.27	31.27	—	PISY, PCAB	71	103	Oleynikov O.M., Dolgova E.A., Matskovsky V.V., Semenyak N.S., Kuznetsova V.V., Pezhemskiy D.V., Solomina O.N.	1149	1814	^28^ [partly published]	RUS350	https://www.ncei.noaa.gov/access/paleo-search/study/36348	0.483	0.208	A	592	7	1.2	
KOSTROMA	Kostroma region	archaeological materials from the city of Kostroma, wooden houses and churches from Kostroma region	57.6	58.2	40.8	41.3	—	PISY, PCAB	66	87	Lazarev A.S., Solomina O.N., Dolgova E.A., Matskovsky V.V., Kuznetsova V.V., Semenyak N.S., Mikhalenko V.N.	1479	1804	Not published	RUS339	https://www.ncei.noaa.gov/access/paleo-search/study/36337	0.521	0.221	A	278	7	2.5	
ZD1	Russia, Tver region, near town Zapadnaya Dvina	Subfossil oaks from river alluvia	56.04	56.41	31.97	32.22	—	QURO	57	121	Khasanov B.F.	572	1382	^14^	RUS387	https://www.ncei.noaa.gov/access/paleo-search/study/36385	0.621	0.239	C	575	63	11.0	
ZD2	Russia, Tver region, near town Zapadnaya Dvina	Subfossil oaks from river alluvia	56.04	56.41	31.97	32.22	—	QURO	34	70	Khasanov B.F.	1346	1762	Not published	RUS388	https://www.ncei.noaa.gov/access/paleo-search/study/36386	0.597	0.239	C	64	14	21.9	
SMOLENSK	Smolensk region, Smolensk city	archaeological materials from the city of Smolensk	54.778	54.778	32.053	32.053	—	PISY	7	14	Solomina O.N., Matskovsky V.V., Pronin G.N.	1387	1624	^36^	RUS356	https://www.ncei.noaa.gov/access/paleo-search/study/36354	0.435	0.213	B	1070	71	6.6	

*COFECHA quality: A - less than 5% of segments with possible problems, B - from 5% to 10% of segments with possible problems, C - more 10% of segments with possible problems.
