# Supplementary material for: Tree-ring data set for dendroclimatic reconstructions and dendrochronological dating in European Russia
Source: Sci Data. 2022 Jun 27;9:367. doi: 10.1038/s41597-022-01456-6 (PMC9237095; doi:10.1038/s41597-022-01456-6)
Supplement: Supplementary file 1 — Supplementary information [file 41597_2022_1456_MOESM1_ESM.docx]

**Supplementary Information**

**Tree-ring data set for dendroclimatic reconstructions and dendrochronological dating in European Russia**

Olga Solomina, Vladimir Matskovsky, Ekaterina Dolgova, Veronika Kuznetsova, Nadezhda Semenyak, Tatiana Bebchuk,
Vladimir Mikhalenko, Alexey Karpukhin, Bulat Khasanov

This file includes:

Supplementary Tables 1, 2

**Supplementary Table 1.** Description of the ‘living-tree’ chronologies. Printed version of online-only Table 1.

| **Site code** | **Location** | **Latitude, degrees N** | **Longitude, degress E** | **H, m** | **Species** | **No. of trees** | **No. of cores** | **Investigatiors** | **First year** | **Last year** | **ITRDB code** | **Mean series inter-correlation (COFECHA)** | **Average mean sensitivity (COFECHA)** |
| --- | --- | --- | --- | --- | --- | --- | --- | --- | --- | --- | --- | --- | --- |
| B36S | Arkhangelsk region, Primorskiy district, Solovetskiye islands | 65,1800 | 35,9700 | 10 | PISY | 17 | 27 | Solomina O.N., Dolgova E.A. | 1549 | 2010 | RUS318 | 0,58 | 0,2 |
| B42S | Arkhangelsk region, Primorskiy district, Solovetskiye islands | 65,1500 | 35,8000 | 10 | PISY | 12 | 23 | Solomina O.N., Dolgova E.A. | 1537 | 2011 | RUS319 | 0,577 | 0,205 |
| B72E | Arkhangelsk region, Primorskiy district, Solovetskiye islands | 65,0906 | 35,6683 | 20 | PCAB | 21 | 42 | Solomina O.N., Dolgova E.A. | 1789 | 2016 | RUS320 | 0,662 | 0,223 |
| B75S | Arkhangelsk region, Primorskiy district, Solovetskiye islands | 65,0392 | 35,6409 | 9 | PISY | 21 | 42 | Solomina O.N., Mikhalenko V.N., Dolgova E.A. | 1840 | 2016 | RUS321 | 0,613 | 0,238 |
| B75E | Arkhangelsk region, Primorskiy district, Solovetskiye islands | 65,0392 | 35,6409 | 9 | PCAB | 20 | 30 | Solomina O.N., Mikhalenko V.N., Dolgova E.A. | 1838 | 2016 | RUS322 | 0,598 | 0,185 |
| I12L | Arkhangelsk region, Pinega district | 64,6948 | 43,1884 | 29 | LASI | 17 | 31 | Solomina O.N., Matskovsky V.V., Dolgikh A.V. | 1670 | 2012 | RUS332 | 0,53 | 0,248 |
| I11S | Arkhangelsk region, Pinega district | 64,5631 | 43,1616 | 90 | PISY | 10 | 17 | Solomina O.N., Matskovsky V.V., Dolgikh A.V. | 1735 | 2012 | RUS331 | 0,506 | 0,205 |
| SHBO | Vologda region, Kirillov district, Shalgo-Boduny forestry | 60,4500 | 38,4600 | 171 | PISY | 10 | 19 | Solomina O.N., Matskovsky V.V. | 1862 | 2009 | RUS354 | 0,566 | 0,196 |
| KOV | Vologda region, Kirillov district, Kovarzino forestry | 60,1600 | 38,8400 | 138 | PISY | 15 | 27 | Solomina O.N., Matskovsky V.V. | 1786 | 2009 | RUS340 | 0,556 | 0,207 |
| KL | Kologriv, Kostroma region, Kologriv district | 58,9820 | 44,3790 | 200 | PCAB | 10 | 21 | Solomina O.N. | 1713 | 2009 | RUS338 | 0,548 | 0,258 |
| K06S | Kologriv, Kostroma region, Kologriv district, near town Kologriv | 58,8661 | 44,2336 | 141 | PISY | 12 | 21 | Solomina O.N. | 1836 | 2012 | RUS333 | 0,475 | 0,226 |
| KS | Kostroma region, Manturocskiy district | 58,1505 | 44,4952 | 100 | QURO | 17 | 30 | Khasanov B.F. | 1923 | 2012 | RUS341 | 0,533 | 0,227 |
| L10S | Tver region, Rameshkovsky district, near Kiverichi village | 57,3458 | 36,6186 | 155 | PISY | 12 | 24 | Solomina O.N. | 1849 | 2014 | RUS344 | 0,552 | 0,242 |
| A05S | Yaroslavl region, Rostov district, Borisoglebsky town | 57,2524 | 39,1637 | 138 | PISY | 13 | 25 | Solomina O.N., Matskovsky V.V. | 1863 | 2013 | RUS315 | 0,531 | 0,232 |
| SHIR | Tver region, Penovskiy district, near Shirkovo village | 57,0491 | 32,7330 | 224 | PISY | 13 | 20 | Solomina O.N. | 1826 | 2010 | RUS355 | 0,593 | 0,25 |
| X01S | Nizhniy Novgorod region, Zelyoniy gorod | 56,5435 | 44,7969 | 108 | PISY | 18 | 33 | Solomina O.N. | 1834 | 2014 | RUS383 | 0,595 | 0,246 |
| X03S | Nizhniy Novgorod region, Semyonovskiy district | 56,5435 | 44,7969 | 108 | PISY | 12 | 23 | Solomina O.N. | 1773 | 2014 | RUS384 | 0,5 | 0,2 |
| L11E | Tver region, Nelidovo district | 56,4580 | 32,9589 | 244 | PCAB | 19 | 38 | Solomina O.N., Matskovsky V.V., Dolgova E.A. | 1845 | 2014 | RUS345 | 0,573 | 0,253 |
| Y01S | Republik of Mariy El, Volzhskiy district | 56,0494 | 48,4617 | 87 | PISY | 24 | 44 | Solomina O.N., Matskovsky V.V., Kuznetsova V.V., Tishin D.V. | 1802 | 2014 | RUS385 | 0,547 | 0,222 |
| T24S | Republic of Tatarstan, Zelenodolskiy district | 55,9090 | 48,7333 | 86 | PISY | 15 | 29 | Solomina O.N., Kuznetsova V.V. | 1749 | 2016 | RUS374 | 0,601 | 0,234 |
| T25S | Republic of Tatarstan, Zelenodolskiy district | 55,9078 | 48,7323 | 79 | PISY | 15 | 27 | Solomina O.N., Kuznetsova V.V. | 1765 | 2016 | RUS375 | 0,632 | 0,273 |
| M13S | Moscow region, Zvenigorod town | 55,7338 | 36,8408 | 167 | PISY | 20 | 35 | Solomina O.N., Matskovsky V.V. | 1763 | 2014 | RUS347 | 0,49 | 0,245 |
| M18E | Moscow region, Zvenigorod district, Zvenigorod biological station | 55,6961 | 36,7280 | 199 | PCAB | 18 | 35 | Solomina O.N., Matskovsky V.V. | 1885 | 2013 | RUS348 | 0,56 | 0,266 |
| T01S | Republic of Tatarstan, Laishevskiy district, near Saraly village | 55,3000 | 49,2600 | 66 | PISY | 15 | 25 | Solomina O.N., Matskovsky V.V., Kuznetsova V.V., Tishin D.V. | 1714 | 2014 | RUS358 | 0,591 | 0,271 |
| T02S | Republic of Tatarstan, Laishevskiy district, near Saraly village | 55,3000 | 49,2600 | 75 | PISY | 16 | 30 | Solomina O.N., Matskovsky V.V., Kuznetsova V.V., Tishin D.V. | 1808 | 2014 | RUS359 | 0,583 | 0,244 |
| M10S | Ryazan region, Spas-Klepiki district, Mesherskiy bor | 55,2372 | 40,0368 | 125 | PISY | 15 | 30 | Solomina O.N. | 1864 | 2014 | RUS346 | 0,5 | 0,231 |
| T19S | Republic of Chuvashia, Alatyrskiy district | 55,0416 | 46,5946 | 87 | PISY | 22 | 47 | Solomina O.N., Kuznetsova V.V. | 1843 | 2015 | RUS371 | 0,548 | 0,203 |
| M19S | Moscow region, Serpukhov district | 54,9123 | 37,6564 | 181 | PISY | 25 | 40 | Solomina O.N., Dolgova E.A. | 1834 | 2014 | RUS349 | 0,596 | 0,23 |
| T15S | Republic of Chuvashiya, Shemurshinskiy district | 54,9000 | 47,5100 | 200 | PISY | 28 | 46 | Solomina O.N., Kuznetsova V.V. | 1819 | 2017 | RUS370 | 0,621 | 0,303 |
| OZER | Moscow region, Kolomenskiy district, near Ozyory town | 54,8577 | 38,5489 | 134 | PISY | 14 | 21 | Solomina O.N. | 1866 | 2010 | RUS352 | 0,575 | 0,244 |
| Z1-2S | Republik of Mordoviya, Temnikovskiy district | 54,7708 | 43,4049 | 180 | PISY | 21 | 39 | Solomina O.N. | 1881 | 2014 | RUS386 | 0,511 | 0,229 |
| PZ | Kaluga region, Dzerzhinskiy district | 54,7323 | 36,0002 | 170 | QURO | 7 | 12 | Khasanov B.F. | 1806 | 2014 | RUS353 | 0,572 | 0,22 |
| F04S | Smolensk region, Ugransky distrikt, Ugra village | 54,6611 | 34,1367 | 195 | PISY | 11 | 19 | Solomina O.N. | 1895 | 2014 | RUS329 | 0,559 | 0,226 |
| KALU | Kaluga region, Kaluga town | 54,5296 | 36,2009 | 183 | PISY | 18 | 34 | Solomina O.N., Matskovsky V.V., Dolgova E.A., Kuznetsova V.V., | 1735 | 2013 | RUS334 | 0,503 | 0,253 |
| T08S | Tatarstan, Bugulminsky district | 54,4268 | 52,7574 | 240 | PISY | 18 | 31 | Solomina O.N., Matskovsky V.V., Kuznetsova V.V., Tishin D.V. | 1809 | 2015 | RUS365 | 0,627 | 0,269 |
| OPP | Kaluga region, Kozelsk district, Optina pustin' monastery | 54,0493 | 35,8307 | 154 | PISY | 12 | 22 | Solomina O.N., Matskovsky V.V., Dolgova E.A. | 1717 | 2010 | RUS351 | 0,526 | 0,264 |
| W01D | Tula region, Schekinskiy disctrict | 53,9884 | 37,2554 | 175 | QURO | 15 | 27 | Solomina O.N. | 1809 | 2014 | RUS382 | 0,553 | 0,218 |
| TZ | Tula region, Schekinskiy disctrict | 53,9781 | 37,1162 | 180 | QURO | 16 | 34 | Khasanov B.F. | 1770 | 2014 | RUS377 | 0,652 | 0,287 |
| H12E | Kaluga region, Kozelsk, near town Kozelsk | 53,9633 | 35,8131 | 166 | PCAB | 13 | 25 | Solomina O.N., Matskovsky V.V., Dolgova E.A., Kuznetsova V.V., | 1910 | 2014 | RUS330 | 0,585 | 0,289 |
| T07S | Samara region, Stavropolskiy district | 53,4400 | 49,7800 | 342 | PISY | 20 | 36 | Solomina O.N., Matskovsky V.V., Kuznetsova V.V., Tishin D.V. | 1786 | 2014 | RUS364 | 0,591 | 0,296 |
| T06S | Samara region, Stavropolskiy district | 53,4064 | 49,9731 | 162 | PISY | 21 | 42 | Solomina O.N., Matskovsky V.V., Kuznetsova V.V., Tishin D.V. | 1828 | 2015 | RUS363 | 0,705 | 0,277 |
| T10S | Penza region, Kuznetskiy district | 53,3633 | 46,8958 | 292 | PISY | 18 | 32 | Solomina O.N., Kuznetsova V.V. | 1799 | 2014 | RUS367 | 0,565 | 0,237 |
| T09S | Penza region, Kuznetskiy district | 53,3211 | 46,8905 | 257 | PISY | 20 | 34 | Solomina O.N., Kuznetsova V.V. | 1788 | 2014 | RUS366 | 0,645 | 0,281 |
| T04S | Orenburg region, Buzulukskiy district | 53,0300 | 52,1100 | 83 | PISY | 11 | 22 | Solomina O.N., Matskovsky V.V., Kuznetsova V.V., Tishin D.V. | 1801 | 2014 | RUS361 | 0,577 | 0,232 |
| T05S | Orenburg region, Buzulukskiy district | 52,9539 | 52,0585 | 79 | PISY | 21 | 41 | Solomina O.N., Matskovsky V.V., Kuznetsova V.V., Tishin D.V. | 1798 | 2015 | RUS362 | 0,675 | 0,271 |
| T23S | Saratov Region, Khvalynskiy district | 52,5800 | 48,0000 | 256 | PISY | 21 | 41 | Solomina O.N., Kuznetsova V.V. | 1905 | 2015 | RUS373 | 0,669 | 0,236 |
| T22S | Saratov Region, Khvalynskiy district | 52,5000 | 48,0400 | 270 | PISY | 19 | 35 | Solomina O.N., Kuznetsova V.V. | 1815 | 2015 | RUS372 | 0,591 | 0,244 |
| T12D | Lipetsk region, Gryazenskiy district | 52,4600 | 39,6900 | 136 | QURO | 12 | 24 | Solomina O.N., Mikhalenko V.N. | 1816 | 2014 | RUS368 | 0,615 | 0,23 |
| T13S | Tambov region, Inzhavinskiy district | 52,3600 | 42,6000 | 128 | PISY | 15 | 27 | Solomina O.N., Mikhalenko V.N. | 1827 | 2015 | RUS369 | 0,667 | 0,308 |
| T03S | Voronezh region, Bobrovsky district, Khrenovskoy forest | 51,2011 | 40,1993 | 94 | PISY | 21 | 36 | Solomina O.N., Matskovsky V.V., Matveev S.M. | 1741 | 2014 | RUS360 | 0,557 | 0,234 |
| DL | Saratov region, Krasnokutskiy district | 50,7299 | 46,6695 | 60 | QURO | 16 | 29 | Khasanov B.F. | 1908 | 2008 | RUS328 | 0,58 | 0,255 |
| V03S | Belgorod region, Novooskolskiy district | 50,6833 | 37,7960 | 100 | PISY | 14 | 22 | Solomina O.N., Matskovsky V.V. | 1790 | 2014 | RUS380 | 0,576 | 0,282 |
| V02S | Belgorod region, Borisovskiy district | 50,6164 | 35,9386 | 148 | PISY | 17 | 33 | Solomina O.N., Matskovsky V.V. | 1901 | 2014 | RUS379 | 0,501 | 0,223 |
| V01D | Belgorod region, Borisovskiy district | 50,6041 | 35,9814 | 165 | QURO | 16 | 30 | Solomina O.N., Matskovsky V.V. | 1732 | 2014 | RUS378 | 0,758 | 0,234 |
| DJ | Krasnodar region, Mostovskiy district | 43,8820 | 40,4678 | 2100 | PISY | 13 | 21 | Grabenko E.A, Kuderina T.M., Kudikov A.V. | 1726 | 2009 | RUS327 | 0,477 | 0,234 |
| D03F | Krasnodarskiy region, Sochi district, Krasnaya Polyana | 43,7186 | 40,2051 | 1806 | FAOR | 11 | 20 | Solomina O.N., Dolgova E.A. | 1680 | 2011 | RUS325 | 0,657 | 0,377 |
| KHTP | Republic of Karachayevo-Cherkessiya , Karachaevskiy district | 43,4303 | 41,7086 | 2285 | PISY | 38 | 70 | Solomina O.N., Dolgova E.A. | 1678 | 2010 | RUS336 | 0,558 | 0,191 |
| KYZ | Republic of Karachayevo-Cherkessiya , Karachaevskiy district | 43,4280 | 41,3035 | 2392 | PISY | 20 | 12 | Solomina O.N., Dolgova E.A. | 1550 | 2007 | RUS343 | 0,625 | 0,206 |
| CHM | Republic of Karachayevo-Cherkessiya , Karachaevskiy district | 43,4043 | 41,3110 | 1714 | PCAB | 16 | 28 | Solomina O.N., Dolgova E.A., Mikhalenko V.N. | 1466 | 2011 | RUS323 | 0,623 | 0,145 |
| ALI | Republic of Karachayevo-Cherkessiya , Karachaevskiy district | 43,2984 | 41,5709 | 1889 | PCAB | 32 | 45 | Solomina O.N., Dolgova E.A., Mikhalenko V.N. | 1790 | 2011 | RUS316 | 0,53 | 0,168 |
| CHS | Republic of Kabardino-Balkariya, Elbrus district | 43,2608 | 42,5189 | 2470 | PISY | 10 | 18 | Solomina O.N., Dolgova E.A. | 1694 | 2011 | RUS324 | 0,484 | 0,139 |
| TERS | Republic of Kabardino-Balkariya, Elbrus district | 43,2380 | 42,5056 | 2470 | PISY | 14 | 24 | Solomina O.N., Dolgova E.A. | 1714 | 2009 | RUS376 | 0,525 | 0,163 |
| KUB | Republic of North Ossetia, Irafskiy district | 42,8944 | 43,5907 | 2356 | PISY | 18 | 29 | Solomina O.N., Dolgova E.A., Matskovsky V.V. | 1733 | 2008 | RUS342 | 0,513 | 0,185 |
| D61S | Republic of North Osetia, Irafskiy district | 42,8548 | 43,7080 | 2015 | PISY | 21 | 38 | Solomina O.N., Dolgova E.A. | 1600 | 2014 | RUS326 | 0,602 | 0,219 |

**Supplementary Table 2.** Description of the ‘historical’ chronologies. Printed version of online-only Table 2.

| **Site code** | **Location** | **Latitude, Southern-most, degrees N** | **Latitude, Northern-most, degrees N** | **Longitude, Western-most, degrees E** | **Longitude, Eastern-most, degrees E** | **Species** | **No. of trees** | **No. of cores** | **Investigatiors** | **First year** | **Last year** | **ITRDB code** | **Mean series inter-correlation (COFECHA)** | **Average mean sensitivity (COFECHA)** |
| --- | --- | --- | --- | --- | --- | --- | --- | --- | --- | --- | --- | --- | --- | --- |
| SOLOVKI | Arkhangelsk region, Solovetskiy archipelago | 64,95 | 65,19 | 35,64 | 36,01 | PISY, PCAB | 75 | 112 | Solomina O.N., Dolgova E.A., Matskovsky V.V., Semenyak N.S., Mikhalenko V.N. | 1185 | 2008 | RUS357 | 0,545 | 0,225 |
| ARKHAN-GELSK | Arkhangelsk region | 63,4 | 64,7 | 37,4 | 43,4 | PISY, PCAB | 45 | 90 | Solomina O.N., Matskovsky V.V., Semenyak N.S. | 1367 | 2020 | RUS317 | 0,492 | 0,209 |
| KARELIA | Republic of Karelia, aroung Onega Lake | 60,8 | 62,72 | 33,06 | 35,27 | PISY, PCAB | 67 | 34 | Karpukhin A.A., Solov'yeva L.N., Chernukh N.B. | 1376 | 1767 | RUS335 | 0,415 | 0,195 |
| KIRILLOV | Vologda region, Kirillov district, Kirillov town | 59,86 | 59,86 | 38,37 | 38,37 | PISY, PCAB | 71 | 138 | Karpukhin A.A., Solov'yeva L.N., Matskovsky V.V., Solomina O.N. | 1085 | 1744 | RUS337 | 0,511 | 0,214 |
| VOLOGDA | Vologda region, Vologda city | 59,22 | 59,22 | 39,89 | 39,89 | PISY, PCAB | 105 | 206 | Solomina O.N., Matskovsky V.V. | 1518 | 1881 | RUS381 | 0,507 | 0,206 |
| NOVGOROD | Novgorod region, Novgorod city | 58,52 | 58,52 | 31,27 | 31,27 | PISY, PCAB | 71 | 103 | Oleynikov O.M., Dolgova E.A., Matskovsky V.V., Semenyak N.S., Kuznetsova V.V., Pezhemskiy D.V., Solomina O.N. | 1149 | 1814 | RUS350 | 0,483 | 0,208 |
| KOSTROMA | Kostroma region | 57,6 | 58,2 | 40,8 | 41,3 | PISY, PCAB | 66 | 87 | Lazarev A.S., Solomina O.N., Dolgova E.A., Matskovsky V.V., Kuznetsova V.V., Semenyak N.S., Mikhalenko V.N. | 1479 | 1804 | RUS339 | 0,521 | 0,221 |
| ZD1 | Russia, Tver region, near town Zapadnaya Dvina | 56,04 | 56,41 | 31,97 | 32,22 | QURO | 57 | 121 | Khasanov B.F. | 572 | 1382 | RUS387 | 0,621 | 0,239 |
| ZD2 | Russia, Tver region, near town Zapadnaya Dvina | 56,04 | 56,41 | 31,97 | 32,22 | QURO | 34 | 70 | Khasanov B.F. | 1346 | 1762 | RUS388 | 0,597 | 0,239 |
| SMOLENSK | Smolensk region, Smolensk city | 54,778 | 54,778 | 32,053 | 32,053 | PISY | 7 | 14 | Solomina O.N., Matskovsky V.V., Pronin G.N. | 1387 | 1624 | RUS356 | 0,435 | 0,213 |
